# Prognostic evaluation of bispectral index in patients following cardiopulmonary resuscitation

**DOI:** 10.3892/etm.2013.884

**Published:** 2013-01-04

**Authors:** HAN LIU, YING LIU, YING XU, YAN XUE

**Affiliations:** 1 Departments of Intensive Care Unit, Nanjing First Hospital, Nanjing Medical University, Nanjing, Jiangsu 210006, China; 2 Emergency Intensive Care, Nanjing First Hospital, Nanjing Medical University, Nanjing, Jiangsu 210006, China

**Keywords:** bispectral index, Glasgow coma scale score, acute physiology and chronic health evaluation II score, SjO_2_, mortality

## Abstract

The aim of this study was to evaluate the prognosis of patients following cardiopulmonary resuscitation in an intensive care unit (ICU) using bispectral index (BIS) monitoring. The study was a prospective comparative study performed at the academic department of an ICU. A total of 33 adults who received cardiopulmonary resuscitation were enrolled and divided into the surviving and non-surviving groups according to their 7-day survival status. During their stay in the ICU, the BIS and arterial oxygen saturation (SaO_2_) levels of all the patients were continuously monitored. The neurological condition of the patients was measured according to the Glasgow coma scale (GCS). Acute physiological and chronic diseases were measured according to the acute physiology and chronic health evaluation II (APACHE II). SaO_2_ was monitored in all patients. The jugular bulb venous oxygen saturation (SjO_2_) levels were continuously monitored in 23 patients and the difference between the SaO_2_ and SjO_2_ values was used to indicate oxygen metabolism in the brain. The variables in the present study were compared between the 2 groups. The correlations between BIS values and GCS or APACHE II scores were analyzed. The BIS values were significantly higher in the surviving group than in the non-surviving group (P<0.01). The difference between the SaO_2_ and SjO_2_ was significant (P<0.01). There was a positive correlation between BIS values and GCS scores (r=0.821, P<0.01) and between BIS values and APACHE II scores (r=0.434, P<0.05). BIS values may be used to predict the post-resuscitative outcome of patients following cardiopulmonary resuscitation.

## Introduction

Bispectral index (BIS) has been suggested as a new method for electroencephalogram (EEG) signal analysis which is able to transfer the mixed information from analysis of the power and frequency of EEG into a numerical output. BIS is expressed as a score between 0 and 100 which reflects the sedation depth and clear-mindedness grade. A score of 100 is entirely clear-minded, whereas decreased scores indicate increased inhibition of the cerebral cortex (1). BIS combines the frequency, power, phase and harmonic wave of EEG and includes a greater amount of original EEG information which rapidly reflects the functional status of the cerebral cortex. Therefore, BIS has been extensively used for judging the depth of anesthesia and consciousness state and has been considered as an index with high sensitivity and accuracy for evaluating the consciousness state of patients (2–6).

BIS has also been used for the diagnosis of brain death and the evaluation of nervous system disease, which expands its clinical applications (7). In 1996, the use of a BIS monitoring instrument in operating rooms was approved by the United States Food and Drug Administration (FDA). Studies have shown that monitoring has been successfully used to assess the depth of sedation and awareness levels of patients in intensive care units (ICUs). Therefore, BIS monitoring may be used for a wide range of clinical applications. Since BIS monitoring has differences between individuals, methods for evaluating the level of the patients’ awareness, predicting the prognosis of severe brain injury patients and diagnosing brain death remain controversial (6,8–10).

Blood from the brain flows mainly through the internal jugular vein and therefore, the jugular bulb venous oxygen saturation (SjO_2_) index represents the cerebral oxygen metabolism level and reflects cerebral circulation and brain function recovery. Certain studies have noted that the difference between the arterial oxygen saturation (SaO_2_) and SjO_2_ (SaO_2_-SjO_2_) may directly reflect the cerebral tissue oxygen consumption situation. Factors which increase cerebral oxygen consumption cause increases in SaO_2_-SjO_2_, while the SaO_2_-SjO_2_ value is decreased by reductions in cerebral oxygen consumption. Therefore, after cardiopulmonary resuscitation, the correlation between SaO_2_ and SjO_2_ may objectively reflect the cerebral oxygen metabolic status of patients. There is a correlation between BIS values and ischemic brain injury and brain edema. The BIS value in patients is consistent with cerebral cortex cell oxygen consumption (11). The present study evaluated the prognosisof patients following acute cardiopulmonary resuscitation by dynamically measuring BIS and SjO_2_ values.

## Materials and methods

### Clinical data

A total of 33 patients were selected, including 20 males and 13 females with an average age of 51.82±18.65 years. Of the 33 patients, there were 16 with coronary atherosclerotic heart disease, 6 with acute exacerbation chronic obstructive pulmonary disease (AECOPD), 4 with uremia, 2 with hypertrophic obstructive cardiomyopathy, 2 with pulmonary infection and 3 with cardiopulmonary arrest of unknown cause. The resuscitation from cardiopulmonary arrest occurred at the hospital for 24 patients and away from hospital for 9 patients. Clinical mortality or brain death after 1 week of therapy were used as the end-point. The criteria of brain death included, i) irreversible coma with clear cause; ii) persisting disappearance of brainstem reflex (complete disappearance of brain function including brainstem function); and iii) unrecoverable respiratory arrest. The exclusion criteria were based on the following: children <1 year old, patients using sedation and muscle relaxants and patients with a previous history of epilepsy. The present study was conducted in accordance with the Declaration of Helsinki and with approval from the Ethics Committee of the Affiliated Nanjing Hospital of Nanjing Medical University. Written informed consent was obtained from the immediate families of all participants.

### BIS monitoring methods

A multi-functional monitor (Intelli Vue MP40; Philips, Hamburg, Germany) equipped with monitor modules for blood pressure, heart rate, SpO_2_ and BIS was used. The monitor module for BIS was obtained from Philips and the BIS sensor with an Aspect 4-electrode from Aspect Medical Systems, Inc. (Newton, MA, USA). All patients were treated by mechanical ventilation and circulatory support immediately after admission or post-cardiopulmonary resuscitation. The BIS values were monitored as soon as the patients circulation and respiration stabilized. The process of BIS monitoring included, i) wiping away grease from the patient’s forehead skin using an alcohol tampon until evaporation; ii) placing the sensor on the level of the lateral canthus; iii) immobilizing BIS electrode patches and tightly pressing for 5 sec to ensure good connections; iv) connecting the BIS electrode, sensor and BIS module; and v) opening the BIS window in the monitor and selecting the sensor display from the installed menu. After an impedance test, the monitor showed a numerical value and graph. When the signal quality index (SQI) was >80% and electromyogram (EMG) was <40, the data was recorded. If the patients were shivering or had other autonomic nerve reactions, the BIS values were unsuitable for the requirements of the study and were not accurate. At this point, the patients were injected intramuscularly with 1.5 mg/kg tramadol and 5 mg droperidole during shivering or hypothermic autonomic nerve reactions until the SQI and EMG values improved. BIS monitoring was avoided during sputum suction, turnover and management of nursing.

### Other indices

The GCS grading of all patients was routinely recorded following admission or post-cardiopulmonary resuscitation. The acute physiology and chronic health evaluation (APACHE) II scores were acquired following admission or cardiopulmonary resuscitation within 24 h. The SaO_2_, SjO_2_ and BIS values were determined simultaneously. In 23 patients, blood was obtained from the internal jugular vein (basilar vein), measured for SjO_2_ and the difference between SaO_2_ and SjO_2_ was calculated.

### Therapy methods

All patients underwent routine cerebral resuscitation in the ICU, including i) mechanical ventilation with a pattern of synchronized intermittent mandatory ventilation and pressure support ventilation (SIMV+PSV), FiO_2_ 40–60% and maintenance of SpO_2_ ≥95%; ii) circulatory support to maintain a mean arterial pressure >80 mmHg and ensure cerebral perfusion; and iii) application of mannitol, albumin for dehydration and cattle encephalon glycoside and ignotin or Xingnaojing (a traditional Chinese medicine) injection for the activation of cerebral metabolism and function. Ice packs or ice blankets were used to lower temperatures to ensure that the patients axillary temperatures did not exceed 35°C. The use of sedative drugs which interfere with BIS was avoided.

### Statistical analysis

Data were presented as the mean ± SD and statistical analysis was performed using the SPSS 13.0 statistical analysis software (SPSS, Inc., Chicago, IL, USA). The Student’s t-test was used for the comparison of the mean of two samples. Pearson correlation analysis was used for rank correlation analysis between BIS and GCS or BIS and APACHE II scores. P<0.05 was considered to indicate a statistically significant difference.

## Results

### Comparison of BIS, SaO_2_, SjO_2_ and SaO_2_-SjO_2_

The data for these parameters are shown in Table I. Blood from 23 of the patients was obtained from the internal jugular vein to measure SjO_2_ and calculate the difference between SaO_2_ and SjO_2_. In 11 cases in the non-surviving group, the SaO_2_ value was 95.97±1.55%. In 12 cases in the surviving group, the SaO_2_ value was 96.25±1.57%. The SaO_2_ was not significantly different between the 2 groups (t=0.512, P>0.05). The SjO_2_ value of patients in the surviving group was 68.84±4.68%, while that of the non-surviving group was 84.98±2.81%. The SjO_2_ of patients in the surviving group was significantly lower than that of the non-surviving group (t=9.909, P<0.01). The SaO_2_-SjO_2_ value of patients in the surviving group was 27.30±5.58%, while that of the non-surviving group was 10.99±2.96%. The SaO_2_-SjO_2_ value of patients in the surviving group was significantly higher than that of the non-surviving group (t=8.633, P<0.01). The BIS value of patients in the surviving group was 61.00±16.68, while that of the non-surviving group was 8.00±10.39. The BIS value of patients in the surviving group was significantly higher than that of the non-surviving group (t=10.870, P<0.01).

### Correlation between BIS values and GCS scores

**Figure 1.**
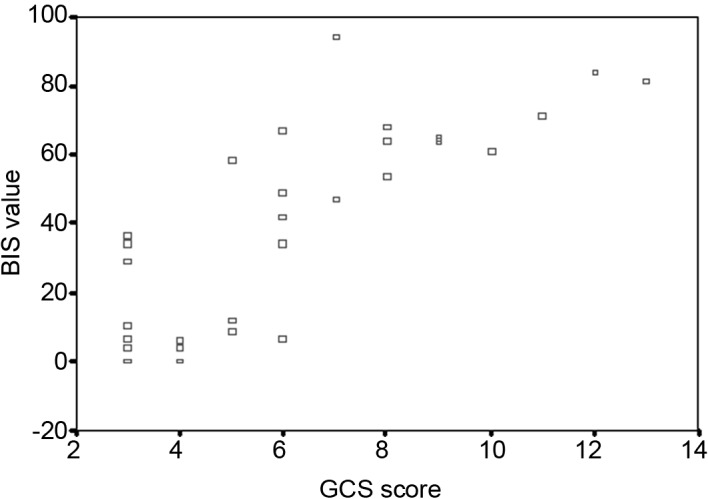
Correlation analysis of BIS value and GCS score. BIS, bispectral index; GCS, Glasgow coma scale.

A scatter diagram of the BIS value and GCS score is shown in Fig. 1. Pearson correlation analysis between BIS and GCS revealed a positive correlation with a correlation coefficient of 0.821 and the association was significant (P<0.001).

### Correlation between BIS values and APACHE II scores

**Figure 2.**
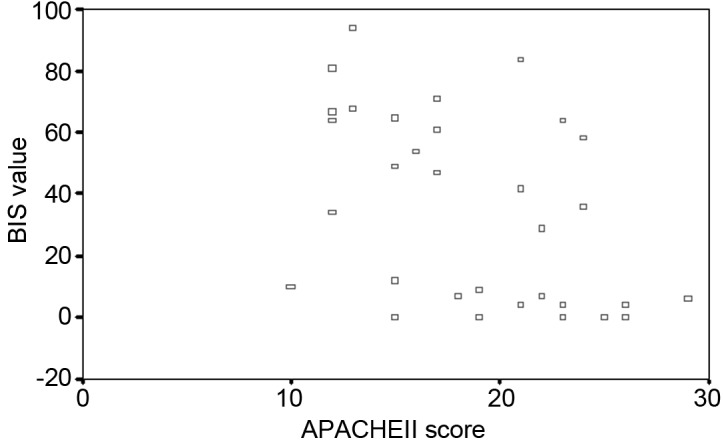
Correlation analysis of BIS value and APACHE II score. BIS, bispectral index; APACHE II, acute physiology and chronic health evaluation II.

A scatter diagram of the BIS value and APACHE II score is shown in Fig. 2. Pearson correlation analysis between BIS and APACHE II revealed a negative correlation with a correlation coefficient of 0.434, and the association was significant (P=0.012).

## Discussion

It has been reported that the EEG shows extensively independent activity ∼10 sec after cardiac arrest. Electrocerebral activity with low voltage and high frequency appears at 15–20 sec after chest cardiac massage (12). Subsequently the electrocerebral activity reverts to normal with the return of the sinus rhythm. Shibata *et al*(13) observed changes in the BIS value of 10 patients following cardiac arrest and noted that the BIS value in patients with successful resuscitation was significantly higher than in those who succumbed. When BIS values are persistently <80, patients enter a vegetative state. Therefore, BIS is considered to predict the prognosis of patients following cardiac pulmonary cerebral resuscitation, although opinions differ (14). Vivien *et al*(15) observed the BIS value and prognosis of 56 patients with severe coma (GCS≤5) and noted that 12 patients with BIS values of 0 were validated to be brain dead via EEG or cerebral angiography. Of the 44 patients with BIS values of between 20 and 79 at admission to the ICU, the BIS value gradually dropped to 0 in 27 patients within several hours or days and progressed to brain death with clinical confirmation. A further 17 patients with an average BIS value >35 did not progress to brain death. The authors proposed that BIS monitoring is useful for predicting the occurrence of brain death in patients with severe coma and provides experimental evidence for the diagnosis of brain death. Fàbregas *et al*(16) reported that BIS values aided the prediction of consciousness recovery when studying cerebral injury.

Analysis of the 33 patients in the present study indicated that the BIS values in the surviving group were significantly higher than those in the non-surviving group. Further analysis revealed that BIS values aided the prediction of the prognosis. When the BIS value was persistently >80, patients were more likely to be clear-minded. When the BIS value was persistently <20 and showed progressive reductions, the patients were unlikely to survive. When the BIS values were persistently stable at 40–60, the majority of patients were in a vegetative state. The results showed that the BIS value was able to predict the prognosis of patients following cardiopulmonary resuscitation. BIS mainly reflects the electrical activity of the cerebral cortex and does not provide any evidence reflecting the electrical activity of the brainstem. In the present study, two patients with BIS values <40, remained alive in a vegetative state. The possible reasons may be the ischemic and hypoxia tolerance time of 4–6 min for the cerebral cortex but 20–30 min for the brainstem (17). Consequently, the respiratory and vasomotor center remained intact and survived in a vegetative state although the BIS values were low in certain patients. The surviving group in the present study included individuals who survived in a vegetative states and others with good recovery of brain function. No subgroup analysis was conducted due to the small number of cases. Enlarging the case number for further subgroup analysis is likely to improve the evaluation of the prognosis.

GCS and APACHE II scores are important indicators for evaluating a patient’s condition in the ICU. It has been reported that there is a good correlation between BIS and GCS (18). In the present study, the BIS value gradually increased with increased GCS scores and the results showed a positive correlation between BIS and GCS. The correlation remained unclear between the BIS and APACHE II score. Although there were correlations between the BIS value and APACHE II score and their association was significant (P<0.05), the low correlation coefficient indicated a weak correlation. The APACHE II score indicates the severity of illness which is mainly associated with the physiological indicators of patients, while GCS scoring reflects the degree of CNS injury. As such, GCS values and APACHE II scores are different indicators. Since BIS reflects the functional status of the cerebral cortex by analyzing the brain’s electrical signals, it may be more closely correlated with the GCS.

It has been reported that BIS values fall to 0 with decreased electrocerebral activity when cardiac arrest results from hypovolemia and rises when the blood pressure increases following blood volume supplementation. The changes to BIS values are delayed by 2 min compared with changes in EEG due to the time required for calculating BIS values via brainwaves. However, changes to BIS occur earlier than hemodynamic changes (19). Besides blood volume, there are several important factors affecting BIS values, including temperature (20,21), instruments in ICUs, levels of blood sugar (22), ethnicity (2), physical therapy (23) and use of muscle relaxant (24–26) or nerve blocker. It is necessary to identify a method for avoiding the signal interference of BIS and provide an improved means of monitoring ICU patients.

The blood circulating in the brain flows through the jugular vein, thus SjO_2_ indicates the level of the cerebral oxygen metabolism and reflects the recovery status of cerebral circulation and function (11). According to the Fick formula (SaO_2_ - SjO_2_ = CMRO_2_ / CBF x CaO_2_), any factor that decreases the consumption of cerebral oxygen decreases the SaO_2_-SjO_2_ value. The normal range of SjO_2_ is 54–75%. When SaO_2_ is at 100%, the normal range of SaO_2_-SjO_2_ is 25–46%. In patients with brain death, the majority or all of the cells in the cerebral cortex are dead and lose the ability to absorb and consume oxygen during the hydrocephalus peak so the SaO_2_-SjO_2_ value is lower than the normal range. BIS may also be low due to the cessation of the brain’s electrical activity. The surviving group in the present study showed higher SaO_2_-SjO_2_ values than the non-surviving group, indicating that cerebral function and metabolism remained present, although there was insufficient perfusion during the hydrocephalus peak and the cells of cerebral cortex were oxygen deficient due to the consumption of oxygen. The BIS value in the surviving group remained higher than that of the non-surviving group. Therefore, there is a association between the changes to the BIS value and the degree of oxygen consumption in cerebral cells. The evaluation of a patient’s prognosis is based on the BIS value which reflects the function of the cerebral cortex. The combination of BIS and SaO_2_-SjO_2_ values may predict the prognosis of a resuscitated patient, but does not change the prognosis of the patient.

An aggressive strategy of cerebral protection is likely to decrease the injury or death of nerve cells, prevent further deterioration and improve the prognosis in patients following cardiac pulmonary resuscitation. The principles of protecting the brain include applying mechanical ventilation, maintaining the correct mean arterial pressure to improve cerebral perfusion and actively lowering the patient’s temperature using an ice pack or blanket. The present study demonstrated that there was a correlation between the changes to the BIS value and the degree of oxygen consumption in cerebral cells which reflected the function of the cerebral cortex. At the the same time as implementing cerebral protection strategies, further studies are required to consider the BIS value as a guide for therapy.

## Figures and Tables

**Table I.** Comparison of BIS, SaO_2_, SjO_2_ and SaO_2_-SjO_2_ in the two groups (mean ± SD).

Group	Case (n)	SaO_2_ (%)	SjO_2_^*^ (%)	SaO_2_-SjO_2_^*^ (%)	BIS value
Non-surviving	16	95.97±1.55	84.98±2.81	10.99±2.96	8.00±10.39
Surviving	17	96.25±1.57	68.84±4.68	27.30±5.58	61.00±16.68
t-value	-	0.512	9.909	8.633	10.870
P-value	-	0.612	<0.01	<0.01	<0.01

* SjO_2_ was measured for 23 of the 33 patients; of these, 11 were in the non-surviving group and 12 were in the surviving group. BIS, bispectral index; SaO_2_, arterial oxygen saturation; SjO_2_, jugular bulb venous oxygen saturation; SaO_2_-SjO_2_, difference between SaO_2_ and SjO_2_.
